# Overnight affective dynamics and sleep characteristics as predictors of depression and its development in women

**DOI:** 10.1093/sleep/zsab129

**Published:** 2021-05-20

**Authors:** Olga Minaeva, Sandip V George, Anna Kuranova, Nele Jacobs, Evert Thiery, Catherine Derom, Marieke Wichers, Harriëtte Riese, Sanne H Booij

**Affiliations:** 1 University of Groningen, University Medical Center Groningen, Department of Psychiatry, Interdisciplinary Center for Psychopathology and Emotion regulation, Groningen, The Netherlands; 2 Department of Psychiatry and Neuropsychology, School of Mental Health and Neuroscience (MHeNS), Maastricht University, Maastricht, The Netherlands; 3 Faculty of Psychology, Open University of the Netherlands, Heerlen, The Netherlands; 4 Department of Neurology, Ghent University Hospital, Ghent University, Ghent, Belgium; 5 Centre of Human Genetics, University Hospital Leuven, KU Leuven, Leuven, Belgium; 6 Department of Obstetrics and Gynecology, Ghent University Hospital, Ghent University, Ghent, Belgium; 7 Lentis, Center for Integrative Psychiatry, Groningen, The Netherlands

**Keywords:** affective inertia, autocorrelation, depression, experience sampling method, negative affect, positive affect, sleep quality, sleep duration

## Abstract

**Study Objectives:**

We examined (1) differences in overnight affective inertia (carry-over of evening affect to the next morning) for positive (PA) and negative affect (NA) between individuals with past, current, and no depression; (2) how sleep duration and quality influence overnight affective inertia in these groups, and (3) whether overnight affective inertia predicts depression development.

**Methods:**

We used data of 579 women from the East-Flanders Prospective Twin Survey. For aim 1 and 2, individuals with past (*n* = 82), current (*n* = 26), and without (lifetime) depression (*n* = 471) at baseline were examined. For aim 3, we examined individuals who did (*n* = 58) and did not (*n* = 319) develop a depressive episode at 12-month follow-up. Momentary PA and NA were assessed 10 times a day for 5 days. Sleep was assessed daily with sleep diaries. Affective inertia was operationalized as the influence of evening affect on morning affect. Linear mixed-effect models were used to test the hypotheses.

**Results:**

Overnight affective inertia for NA was significantly larger in the current compared to the non-depressed group, and daytime NA inertia was larger in the past compared to the non-depressed group. Overnight NA inertia was differently associated with shorter sleep duration in both depression groups and with lower sleep quality in the current compared to the non-depressed group. Overnight affective inertia did not predict depression development at 12-month follow-up.

**Conclusions:**

Current findings demonstrate the importance of studying complex affect dynamics such as overnight affective inertia in relation to depression and sleep characteristics. Replication of these findings, preferably with longer time-series, is needed.

**Statement of Significance**

Momentary affect fluctuations of an individual are nowadays easily assessed by filling out questionnaires multiple times a day on a smartphone. This is relevant for depressed individuals; carry-over effects of affective states from one moment to the next are stronger in depressed compared to non-depressed individuals. However, carry-over effects from evening to morning are understudied. In a female general population sample, we found that overnight carry-over effects were stronger for negative affect in individuals with current and past depression, but not in those who developed depression over the next 12-months. Moreover, poor and short sleep was related to differences in carry-over effects between the groups. To make progress in the field, findings need to be replicated in longer time-series data.

## Introduction

Depression is a complex disorder with a multi-factor and not fully understood etiology, which motivates researchers to investigate its mechanisms and features. Approximately 15% of people suffer from depressive disorders at some point in their lives [1]. With their high prevalence, depressive disorders are among the leading causes of non-fatal health loss globally for nearly three decades and are associated with a high burden for those suffering from it, their family, and society [2]. Therefore, insight in possible mechanisms is crucial for better understanding of etiology of depression.

One of the depression features is a disturbance in negative (NA) and positive affect (PA) fluctuations, specifically the “stickiness” of affective states, also termed emotional or affective inertia [3]. Affective inertia refers to the degree to which an individual’s current affective state is predicted by their affective state at the prior time point and is often operationalized as the autocorrelation of an affective state. It reflects how much affect carries over from one moment to the next. Consequently, individuals with low affective inertia recover more quickly from an emotional disturbance than individuals with high affective inertia [4]. There are multiple studies to date that have investigated within-person affective inertia throughout the day in relation to depression. Their findings show that having an emotional system that quickly regulates emotions back to baseline, is adaptive and important for keeping mental health [4]. Increased negative affective inertia (i.e. high autocorrelation of NA) has been reported for depressed compared to non-depressed individuals [5]. It was also shown to have the important predictive role for the development of future depressive symptoms [6, 7]. Conversely, increased positive affect inertia, i.e. the ability to transfer PA from one moment to the next, seems to be an important factor in both prevention of, and recovery from depressive symptoms [8].

Opposed to affective inertia during the daytime, the role of affective inertia from the evening to the next morning in depression, and the role of sleep therein, has been scarcely studied. This is surprising considering the protective role that good sleep plays in human emotional homeostasis and regulation [9] and the often-reported dysregulated sleep in depressed individuals [10–13]. In clinical samples, insomnia has been reported in about 75% of all depressed patients. In epidemiological samples, the incidence of sleep disturbances varied from 50% in young adults up to 90% in older adults [14]. Experimental evidence suggests that a good night sleep helps individuals regulate their emotions back to their baseline values [15]. On a neurobiological level, poor sleep seems to prohibit individuals from “resetting the correct affective brain reactivity” to emotional challenges the next day (by restoring functional connectivity between the amygdala and the medial prefrontal cortex) [16]. In addition, an observational study among adolescents examined the role of sleep duration on overnight affective inertia of daily stress-related affect (referred in the study as spillover effects) [17]. On average, small and non-significant stress-related affective spillover effects were found. However, spillover effects were more pronounced and significant in those individuals with shorter nighttime sleep duration. Hence, both neurobiological and psychological studies support the relevance of the concept of overnight affective inertia, and that poor sleep is related to increases in it.

Although direct evidence for a link between overnight affective inertia and depression is lacking, more indirect evidence comes from various observational studies comparing both depressed and non-depressed individuals on the relationship between sleep and next day’s affect as well as between affect and the following night sleep. In a sample of depressed and non-depressed individuals, worse sleep quality predicted next day’s lower PA and higher NA regardless of depression status [18]. In another study, the effect of sleep quality on next day’s NA and PA did not differ for different levels of depressive symptoms [19]. Yet, another group of researchers did find that levels of depressive symptoms influence the effect of sleep quality on NA but not PA and not the effects of PA or NA on sleep [20]. They found that depressed individuals deteriorate more from poor sleep quality than non-depressed in terms of their morning affect [20]. However, whether this also applies for overnight affective inertia remains unclear.

In this study, we aimed to address the gaps mentioned above by examining overnight affective inertia in a longitudinal study including individuals with depression (both past and current depression diagnosis) and individuals without a history of depression.

1. We examined whether there is a difference in overnight affective inertia between (a) currently depressed and never depressed individuals, and (b) individuals with a history of depression and never depressed individuals.

We hypothesized that currently depressed individuals and those with a history of depression would have increased overnight affective inertia (i.e. higher autocorrelation) than never depressed individuals.

2. We investigated how sleep characteristics influence overnight affective inertia, and whether this differs between (a) currently depressed and never depressed individuals and (b) individuals with a history of depression and never depressed individuals.

We expected larger overnight affective inertia in the depression groups in case of poor sleep quality and shorter sleep duration, and similar affective inertia between the groups with good sleep quality and sufficient sleep duration. As prior studies often included either sleep quality or duration to examine associations with affect, and it is not well known how these two measures compare, we included both in our study. Hence, from that perspective, depressed individuals could be more susceptible to the detrimental effects of poor sleep quality or shorter sleep duration.

3. We explored if differences in affective inertia were associated with the development of depression at 12-months follow-up.

We hypothesized that overnight affective inertia will be larger at baseline in individuals who developed a depressive episode at the 12-months follow-up assessment. As previous studies reported predictive capacity of daytime affective inertia for future depression, we intended to test this association for overnight affective inertia.

4. Finally, to replicate earlier findings on daytime affective inertia, and to compare overnight and daytime affective inertia for the first time, we examined associations with daytime affective inertia as well.

## Methods

### Study population

We used a sample of 621 female participants from the East-Flanders Prospective Twin Survey in Belgium—a general population sample from birth registers of Flemish municipalities—who filled out ESM questionnaires for 5 days (details in [21, 22]). Of the 621 participants, 610 completed the ESM measurements. Thirty-one were excluded due to inadequate ESM compliance, resulting in a final sample of 579 participants. Participants were categorized into three groups at baseline: currently depressed individuals (*n* = 26), individuals with a history of depression but not currently depressed (*n* = 82), and never depressed individuals (*n* = 471). To answer the third, prospective, research question, the participants were categorized into two new groups: individuals who developed a depressive episode between baseline and 12 months follow-up (*n* = 58) and non-depressed individuals at the follow-up (*n* = 359). From those who developed depression, 48 participants developed it during the 12 months between baseline and follow-up assessment; 10 participants developed a depression at the time of the 12-months follow-up assessment. The sample for the last hypothesis became noticeably smaller after we excluded individuals with a missing diagnosis at the follow-up (*n* = 136) and individuals with current depression at baseline (*n* = 26). Currently depressed individuals were excluded since the measurements of their affective inertia could be attributed to the current depression episode rather than depression developed during the follow-up what could potentially bias the analyses. All participants gave written informed consent after Ethics Committee approval.

### Measurements

#### ESM assessment

Participants received a digital wristwatch and a set of ESM self-assessment forms collated in a booklet for each day. The wristwatch was programmed to emit a signal (beep) at an unpredictable moment in each of ten 90-min time blocks between 07:30 am and 22:30 pm on five consecutive days. After each beep, participants were required to stop their activity and fill out the ESM self-assessment forms.

Selection of the ESM affect items was based on results of previous ESM studies (selecting items with high loadings on NA and PA latent factors and sufficient within-person variability) [23]. PA was assessed at each beep with four mood adjectives (I feel “cheerful,” “content,” “energetic,” and “enthusiastic”) rated on seven-point Likert scales as described above. Since our data has a multilevel structure, we applied a composite reliability index (omega) to account for level-specific reliability [24]. The mean of the four items formed the PA scale; within-person omega was 0.79 and between-person omega was 0.95. NA was assessed with six mood adjectives (I feel “insecure,” “lonely,” “anxious,” “low,” “guilty,” and “suspicious”) and within-person omega was 0.68 and between-person omega was 0.91. For the analysis, we used weighted PA and NA scores to account for missing values and secure equal representation of different subjects in sum scores.

#### Depression diagnosis and symptomology

The Structured Clinical Interview for DSM-IV Axis I Disorders (SCID-I; [25]) was administered to obtain current and lifetime diagnosis of major depressive disorder. To assess depressive symptoms, the depression subscale on the SCL-90-R was used [26]. Participants rated each item on a five-point scale from 0 (not at all) to 4 (extremely) specifying how much each has bothered them during the past 7 days. Participants were assessed with the diagnostic interview at the baseline and at the 12-month follow-up assessment, and with the SCL-90 at each of the five waves (baseline, 3-, 6-, 9-, and 12-months follow-up).

#### Sleep

Subjective sleep was assessed with a sleep diary. A sleep diary is a way of prospectively self-monitoring sleep and is regarded as the gold standard for subjective sleep assessment [27]. Average time between waking up and the first beep was 51 min. Each morning, participants rated their sleep quality pertaining to the previous night on a 7-point Likert scale ranging from 1 (not at all) to 7 (very well), sleep onset latency (time taken to fall asleep, in minutes), and number of awakenings. Furthermore, participants reported the (clock-) time of awakening and getting up, as well as their bedtime, enabling estimation of total time in bed (minutes between time to bed and getting up). In order to estimate total sleep duration, the variable sleep period was calculated as the minutes between reported sleep onset and awakening.

### Statistical analysis

The hypotheses were investigated using linear mixed-effect models, which generalizes linear models to account for the hierarchical structure in data. Such an approach offers multiple advantages including being able to handle missing data efficiently and accounting for clustering in data [28, 29]. Our data had three levels; observations (level 1) were clustered within individuals (level 2) who (sometimes) belonged to a twin pair (level 3).

To distinguish within-person from between-person effects, all time varying predictors in our models were separated into a person-mean and a person-mean centered variable. This was done by calculating the mean of the individual (person-mean variable) and subtracting it from the original series (person-mean centered variable). On the individual level, a random intercept and a random slope for the affect variables were added to correct for the different mean levels of affect and for possible individual trends in affect. On the twin level, a random intercept was added to account for within-twin pair covariances. Model fit was assessed using the Akaike Information Criterion (AIC) (lower is better). Since an unstructured covariance matrix led to convergence problems, a diagonal structure was chosen for the random effects’ covariance matrix instead. All analyses were conducted using the *nlme* package in R version 3.6.1.

Overnight affective inertia was operationalized in the analyses as the effect of the last measurement of the previous day evening affect (evening affect_*t−*1_) on the first measurement of current day’s morning affect (morning affect_t_) over 5 days. Affective inertia during the day was operationalized as the effect of affect_*t−*1_ on adjacent time point affect_t_. The different models used for testing our hypotheses will be described for the four outcome variables at once (NA_t_ and PA_t_ in the morning and during the day), and any differences will be mentioned. All significant two- or three-way interactions of p<0.05 were analyzed stratified and further inspected using plots. To correct for multiple testing, we performed the Benjamini–Hochberg FDR-controlling (BH) procedure with a false discovery rate of 25% [30].

For the first hypothesis, we examined whether overnight affective inertia was significantly different between the three groups by assessing the effect of the interaction between person-mean centered evening affect_*t−*1_ and dummy-coded variables for current and past depression, on morning affect_t_ (Model 1a). In a second step, this association was tested while including person-mean and person-mean centered sleep quality and sleep duration variables as controlling factors (Model 1b), because sleep variables are known to significantly influence morning affect. For day time affective inertia, the same model was used (Model 1a) to study affect_*t−*1_ predicting affect_*t*_.

For the second hypothesis, we examined the association between sleep quality and overnight affective inertia, and whether it differed according to depression status. To accomplish this, morning affect was regressed on the three-way interaction between evening affect_*t−*1_, person mean sleep quality, and dummy-coded variables for current and past depression (Model 2a). If there was no significant three-way interaction effect found, it was removed in the next step, while retaining the two two-way interaction terms between evening affect and sleep quality and between sleep quality and the dummy-coded depression variables (Model 2b). The effect of sleep duration on overnight affective inertia was examined the same way. If two-way interaction effects for sleep quality and sleep duration were significant, they were subsequently assessed in the same regression model (Model 2c), since one significant two-way interaction may be explained by the other, and vice versa.

For the third hypothesis, we investigated how overnight affective inertia at baseline predicts the development of depression during the 12-months follow-up, in the subsample of non-depressed individuals at baseline. In order to investigate this question, we constructed a multilevel model, inverting the predictor and outcome variables. Hence, we examined how the development of depression predicts the morning affect, and how it interacts with the evening affect. The (morning) affect_*t*_ was regressed on the two-way interaction between a dummy-coded variable for depression at follow-up and (evening) affect_*t−*1_. Having a depression episode in the past could potentially have lasting influence on affective inertia and serve as a vulnerability factor for future depression. Hence, the model was controlled for past depression at baseline. In case of any significant interaction or main effects, a sensitivity check would be performed to take into account individual differences in depressive symptom levels at baseline. To be able to test the third hypothesis more directly, we also implemented a two-step model as a sensitivity analysis. First, we extracted random effects (Best Linear Unbiased Estimates) from a multilevel model for overnight affective inertia (a simple model without moderators or covariates) and then used these as a predictor in a univariate model, with depression status at 12 months as an outcome in a separate analysis [31]. This also allowed us to control for baseline depressive symptom levels, as the groups with and without a developed depression at follow-up differed in this respect. A detailed description of the models used to test each hypothesis can be found in Supplementary Material 1. Results for the sensitivity analysis are given in Supplementary Table S1.

## Results

Demographic and psychopathology characteristics of three baseline subgroups and two follow-up subgroups (current, past, and never depressed groups at baseline and development of depression and non-depressed groups at 12-months follow-up) are presented in Table 1. The mean SCL-90 scores of the whole sample at baseline were as follows: 1.45 for depression subscale, 1.38 for anxiety subscale, 1.42 for paranoid ideation subscale, and 1.14 for psychoticism subscale. The mean SCL-90 depression score at baseline was significantly higher for the group of individuals who developed depression at 12-months follow-up compared to the group of individuals who did not.

**Table 1.** Characteristics of baseline (*n* = 579) and follow-up (*n* = 417) groups of the dataset

	Baseline groups			Follow-up groups	
Characteristic	Past depression	Current depression	Never depressed	Developed depression	Non-depressed
Group size, *n* (%)	82 (14.20)	26 (4.50)	471 (81.30)	58 (13.90)	359 (86.10)
Age, mean (*SD*)	26.93 (7.30)	28.23 (8.69)	32.11 (9.41)	29.41 (7.34)	26.89 (7.25)
Education, *n* (%)					
Primary school	1 (1.20)	1 (3.80)	2 (0.40)	1 (1.70)	2 (0.60)
Secondary education	33 (40.20)	11 (42.30)	162 (34.40)	26 (44.80)	122 (34.00)
College or university degree	46 (56.10)	13 (50.00)	301 (64.00)	31 (53.40)	231 (64.30)
Employment, *n* (%)					
Employed	55 (67.10)	14 (53.80)	285 (60.80)	39 (67.20)	210 (58.50)
Student	21 (25.60)	11 (42.30)	173 (36.90)	14 (24.10)	144 (40.10)
Unemployed	5 (6.10)	1 (3.80)	9 (1.90)	4 (6.90)	5 (1.40)
Other	1 (1.20)	0 (0)	2 (0.40)	1 (1.70)	0 (0)
Marital status, married, *n* (%)	39 (47.60)	6 (23.10)	158 (33.50)	21 (36.20)	127 (35.40)
SCL-90-R score at baseline per subscale					
Depression subscale, mean (*SD*)	1.64 (0.58)	2.28 (0.92)	1.37 (0.41)	1.67 (0.62)	1.35 (0.39)
Anxiety subscale, mean (*SD*)	1.54 (0.59)	1.99 (0.63)	1.32 (0.38)	1.54 (0.57)	1.31 (0.37)
Paranoid ideation subscale, mean (*SD*)	1.68 (0.70)	1.96 (0.74)	1.35 (0.44)	1.62 (0.60)	1.35 (0.44)
Psychoticism subscale, mean (*SD*)	1.24 (0.42)	1.41 (0.40)	1.11 (0.18)	1.23 (0.31)	1.09 (0.16)
Daily NA, median (*SD*)	0.74 (0.36)	0.80 (0.47)	0.71 (0.25)	0.79 (0.32)	0.70 (0.24)
Daily PA, mean (*SD*)	3.48 (0.83)	3.18 (0.86)	3.57 (0.78)	3.45 (0.75)	3.59 (0.80)
Wake up time, mean (*SD*)	8:09 (1:38)	8:04 (1:36)	8:07 (1:28)	8:07 (1:31)	8:06 (1:27)
Time of going to sleep, median (*SD*)	23:28 (1:31)	23:24 (1:20)	23:31 (1:26)	23:33 (1:28)	23:30 (1:26)
Sleep duration, h, mean (*SD*)	8.25 (1.53)	8.02 (1.73)	8.17 (1.47)	8.13 (1.40)	8.15 (1.47)
Sleep quality, mean (*SD*)	5.04 (1.56)	4.50 (1.55)	5.30 (1.43)	4.82 (1.62)	5.32 (1.43)

Abbreviations: SCL-90–R = The Symptom Checklist-90 Revised; NA = negative affect; PA = positive affect.

Notes. The time variables (wake up time, time of going to sleep) are given in 24-h clock time.

### Research question 1: The association between depression status and overnight and daytime affective inertia

The results from linear mixed-effects models estimating associations between morning and preceding evening affect (overnight affective inertia) and the group status for PA and NA are given in Table 2. We found a significant moderation effect with currently depressed individuals having significantly higher overnight negative affective inertia compared to never depressed ones (*B* = 0.509). No significant moderation effects were found for PA and for NA and past depression compared to never depressed.

**Table 2.** Linear mixed-effects models estimated associations between morning and preceding evening affect (overnight autocorrelation) for negative affect (NA) and positive affect (PA) in past and current depression groups compared to never depressed groups

	Negative affect			Positive affect		
Fixed effects	*B*	95% CI	*p*-value	*B*	95% CI	*p*-value
***Overnight affective inertia***						
*Model 1a. Assessing differences between groups in overnight affective inertia* (*n* = 1,312)*						
Intercept	**0.797**	**0.770; 0.824**	**<0.001**	**3.456**	**3.368; 3.543**	**<0.001**
Evening Affect (autoregressive effect)	**0.168**	**0.050; 0.287**	**0.005**	**0.299**	**0.216; 0.382**	**<0.001**
Depression Group (ref = never depressed)						
Past depression	**0.082**	**0.011; 0.152**	**0.024**	0.009	−0.208; 0.225	0.937
Current depression	**0.196**	**0.082; 0.311**	**<0.001**	−**0.437**	−**0.782;** −**0.093**	**0.013**
Evening affect × depression group (ref = never depressed)						
Evening affect × past depression	0.231	−0.058; 0.520	0.117	0.111	−0.085; 0.308	0.266
Evening affect × current depression	**0.509**	**0.101; 0.916**	**0.015**	0.079	−0.264; 0.422	0.651
*Model 1b. Assessing differences between groups in morning affect* (*n* = 1,312)						
Intercept				**3.456**	**3.368; 3.543**	**<0.001**
Evening affect				**0.322**	**0.249; 0.395**	**<0.001**
Depression group (ref = never depressed)						
Past depression				0.008	−0.209; 0.224	0.943
Current depression				−**0.438**	−**0.782;** −**0.093**	**0.013**
*Model 1c. Assessing differences between groups in overnight affective inertia, controlling for sleep* (*n* = 876)						
Intercept	**1.017**	**0.811; 1.224**	**<0.001**	**3.113**	**2.498; 3.729**	**<0.001**
Evening affect (autoregressive effect)	0.136	−0.027; 0.298	0.101	**0.336**	**0.229; 0.444**	**<0.001**
Depression group (ref = never depressed)						
Past depression	0.032	−0.049; 0.113	0.439	0.009	−0.235; 0.252	0.943
Current depression	**0.220**	**0.085; 0.354**	**0.002**	−0.320	−0.718; 0.078	0.115
Person-mean sleep quality	−**0.046**	−**0.068;** −**0.023**	**<0.001**	**0.170**	**0.103; 0.236**	**<0.001**
Person-mean centered sleep quality	−0.016	−0.038; 0.005	0.142	**0.069**	**0.015; 0.124**	**0.012**
Person-mean sleep duration	0.004	−0.018; 0.025	0.749	−**0.068**	−**0.132;** −**0.004**	**0.038**
Person-mean centered sleep duration	0.001	−0.018; 0.021	0.888	0.043	−0.007; 0.092	0.089
Evening affect × depression group (ref = never depressed)						
Evening affect × past depression	0.297	−0.149; 0.742	0.191	0.068	−0.206; 0.343	0.625
Evening Affect * Current depression	0.566	−0.024; 1.157	0.060	0.214	−0.185; 0.613	0.292
*Model 1d. Assessing differences between groups in morning affect, controlling for sleep* (*n* = 876)						
Intercept	**1.018**	**0.811; 1.224**	**<0.001**	**3.112**	**2.496; 3.728**	**<0.001**
Evening affect	**0.209**	**0.066; 0.353**	**0.004**	**0.360**	**0.265; 0.454**	**<0.001**
Depression group (ref = never depressed)						
Past depression	0.032	−0.050; 0.113	0.442	0.009	−0.235; 0.252	0.944
Current depression	**0.219**	**0.084; 0.354**	**0.002**	−0.322	−0.720; 0.076	0.112
Person-mean sleep quality	−**0.046**	−**0.068;** −**0.023**	**<0.001**	**0.170**	**0.103; 0.236**	**<0.001**
Person-mean centered sleep quality	−0.016	−0.037; 0.006	0.159	**0.068**	**0.014; 0.122**	**0.014**
Person-mean sleep duration	0.004	−0.018; 0.025	0.748	−**0.068**	−**0.132;** −**0.004**	**0.038**
Person-mean centered sleep duration	0.002	−0.017; 0.022	0.825	0.040	−0.009; 0.089	0.107
***Daytime affective inertia***						
*Model 1e. Assessing differences between groups in daytime affective inertia* (*n* = 20,027)						
Intercept	**0.825**	**0.800; 0.849**	**<0.001**	**3.583**	**3.512; 3.655**	**<0.001**
Affect_ t_− _1_ (autoregressive effect)	**0.185**	**0.159; 0.211**	**<0.001**	**0.236**	**0.215; 0.256**	**<0.001**
Depression group (ref = never depressed)						
Past depression	**0.097**	**0.035; 0.160**	**0.002**	−0.065	−0.231; 0.100	0.438
Current depression	**0.209**	**0.106; 0.311**	**<0.001**	−**0.457**	−**0.727;** −**0.187**	**0.001**
Affect_*t*_− _1_ × depression group (ref = never depressed)						
Affect_*t*_− _1_ × past depression	**0.067**	**0.005; 0.129**	**0.035**	0.016	−0.035; 0.067	0.545
Affect_*t*_− _1_ × current depression	0.048	−0.052; 0.148	0.346	0.004	−0.080; 0.088	0.928
*Model 1f. Assessing differences between groups in affect at the previous time point* (*n* = 20,027)						
Intercept				**3.583**	**3.512; 3.655**	**<0.001**
Affect_*t*_− _1_				**0.238**	**0.220; 0.256**	**<0.001**
Depression group (ref = never depressed)						
Past depression				−0.065	−0.231; 0.100	0.438
Current depression				−**0.457**	−**0.727;** −**0.187**	**0.001**

Notes. All significant effects related to hypotheses survived the Benjamini–Hochberg FDR-controlling (BH) procedure with a false discovery rate of 25%. Model 1a includes person-mean centered PA or NA in the evening of the previous day and interaction between person-mean centered PA or NA in the evening of the previous day and the group status (past or current depression), where the non-depressed group is the reference group. Model 1c includes person-mean centered PA or NA in the evening of the previous day, person-mean sleep quality/duration, person-mean centered (deviation from a person mean) sleep quality/duration, and interaction between person-mean centered PA or NA in the evening of the previous day and the group status. For daytime affective inertia, Model 1e includes person-mean centered PA or NA at a previous time point (Affect_*t*_− _1_) instead of affect in the evening of the previous day. All models include the intercept.

*n = number of observations included in each model.

After controlling for sleep variables, the moderation effect of current depression, although increased (*B* = 0.566), became non-significant (*p* = 0.060) (Model 1c). However, it should be noted that there was a substantial decrease in observations in these controlled analyses, particularly for the one with sleep duration (from 1312 to 876 observations).

**Figure 1.**
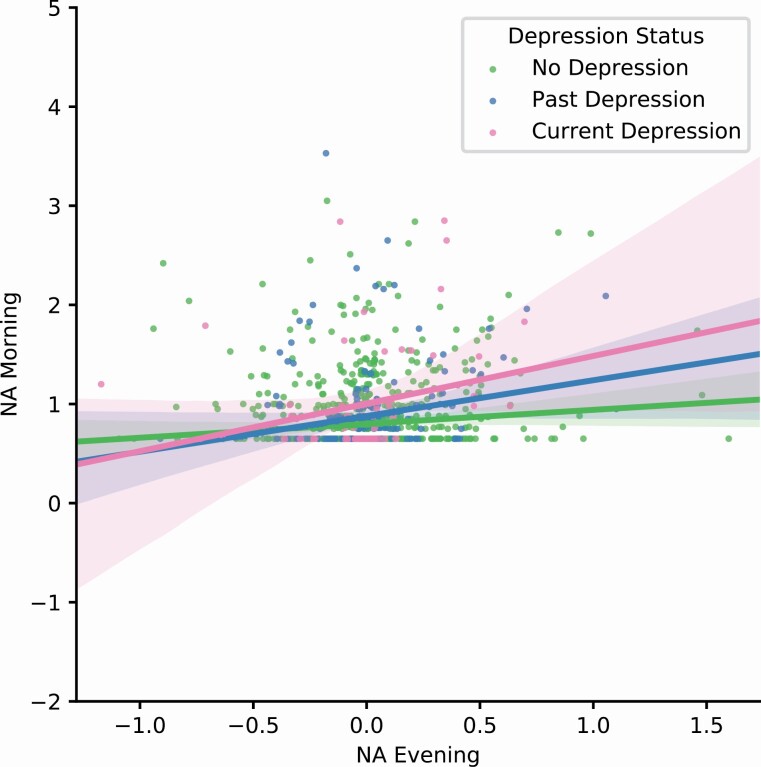
Overnight affective inertia for negative affect (NA) for current, past, and never depressed groups.

We performed a subgroup analysis and plotted the association between evening NA and morning NA separately for the three groups (Figure 1). The subgroup analysis revealed significant associations between evening NA and morning NA in each group separately, which remained significant only in the current and past depression groups after controlling for sleep quality and duration (Supplementary Table S1). Thus, higher evening NA was associated with higher morning NA in both depression groups but not in the never depressed group.

Controlling for sleep variables did not change the moderation effect for the depression groups for PA (Model 1c). After removal of the non-significant interaction with depression status (AIC changed from 2393 to 2387), overnight affective inertia for PA was assessed (Models 1b and 1d), and morning PA was predicted by the evening PA_*t−*1_.

For daytime affective inertia (*research question 4*), we found a small but significant moderation effect of past depression on the effect of the previous time point NA_*t−*1_ but not PA_*t−*1_ on the next time point NA_t_ and PA_t_, respectively (Model 1e). This means that individuals in the past depression group had increased levels of negative affective inertia (affective inertia of NA) during the day, compared to never depressed individuals (Supplementary Figure S1).

After removal of the non-significant interactions between PA_*t−*1_ and PA_*t*_, affective inertia was much smaller during the day (*B* = 0.238) compared to overnight affective inertia for PA (*B* = 0.360) (Models 1d and 1f).

### Research question 2: The association between sleep characteristics (duration and quality) and overnight affective inertia in three groups

Table 3 provides the results for the three-way interaction between evening PA_*t−*1_ and NA_*t−*1_, sleep characteristics and depression status. We found significant moderation effects between evening NA, sleep quality and current depression and between evening NA, sleep duration and both current and past depression. This means that the size of overnight negative affective inertia was differently associated with these sleep characteristics in the different depression groups (Models 2a and 2b).

**Table 3.** Linear mixed-effects models estimating associations between morning affect, preceding evening affect (overnight autocorrelation), sleep measures and group status for negative affect (NA) and positive affect (PA)

	Negative affect			Positive affect		
Fixed effects	*B*	95% CI	*p*-value	*B*	95% CI	*p*-value
*Model 2a. Assessing differences between groups in overnight affective inertia, moderated by sleep quality* (*n* = 1,273)*						
Intercept	**1.064**	**0.936; 1.191**	**<0.001**	**2.542**	**2.162; 2.923**	**<0.001**
Evening affect (autoregressive effect)	−0.177	−0.723; 0.370	0.526	**0.489**	**0.045; 0.933**	**0.031**
Depression group (ref = never depressed)						
Past depression	0.088	−0.230; 0.405	0.587	−0.147	−1.079; 0.784	0.756
Current depression	**0.819**	**0.340; 1.297**	**<0.001**	−**1.723**	−**3.163;** −**0.282**	**0.019**
Sleep quality	−**0.049**	−**0.072;** −**0.026**	**<0.001**	**0.170**	**0.101; 0.240**	**<0.001**
Evening affect × depression group (ref = never depressed)						
Evening affect × past depression	1.286	−0.126; 2.699	0.074	−0.072	−1.160; 1.015	0.896
Evening affect × current depression	**3.333**	**0.924; 5.742**	**0.007**	−1.641	−3.672; 0.390	0.113
Evening affect × sleep quality	0.067	−0.038; 0.172	0.212	−0.035	−0.118; 0.047	0.399
Depression group (ref = never depressed) × sleep quality						
Past depression × sleep quality	−0.004	−0.064; 0.057	0.909	0.036	−0.142; 0.214	0.690
Current depression × sleep quality	−**0.148**	−**0.251;** −**0.045**	**0.005**	**0.318**	**0.009; 0.627**	**0.044**
Evening affect × depression group (ref = never depressed) × sleep quality						
Evening affect × past depression × sleep quality	−0.208	−0.491; 0.075	0.149	0.033	−0.167; 0.233	0.744
Evening affect × current depression × sleep quality	−**0.669**	−**1.239;** −**0.099**	**0.022**	0.382	−0.078; 0.842	0.103
*Model 2b. Assessing differences between groups in overnight affective inertia, moderated by sleep duration* (*n* = 874)						
Intercept	**0.717**	**0.514; 0.920**	**<0.001**	**4.007**	**3.393; 4.621**	**<0.001**
Evening affect (autoregressive effect)	−**1.513**	−**2.736;** −**0.291**	**0.015**	0.466	−0.320; 1.252	0.244
Depression group (ref = never depressed)						
Past depression	0.545	−0.006; 1.095	0.053	−1.245	−2.879; 0.389	0.135
Current depression	**1.189**	**0.485; 1.893**	**0.001**	1.081	−1.001; 3.164	0.307
Sleep duration	0.011	−0.014; 0.036	0.396	−0.067	−0.143; 0.008	0.081
Evening affect × depression group (ref = never depressed)						
Evening affect × past depression	**4.260**	**0.406; 8.114**	**0.030**	−0.028	−2.162; 2.106	0.980
Evening affect × current depression	**4.923**	**0.906; 8.940**	**0.016**	3.634	−0.823; 8.092	0.110
Evening affect × sleep duration	**0.206**	**0.055; 0.358**	**0.008**	−0.016	−0.113; 0.080	0.739
Depression group (ref = never depressed) × sleep duration						
Past depression × sleep duration	−0.064	−0.132; 0.004	0.064	0.150	−0.051; 0.351	0.143
Current depression × sleep duration	−**0.117**	−**0.204;** −**0.030**	**0.008**	−0.193	−0.451; 0.064	0.140
Evening affect × depression group (ref = never depressed) × sleep duration						
Evening affect × past depression × sleep duration	−**0.483**	−**0.936;** −**0.031**	**0.036**	0.010	−0.252; 0.271	0.941
Evening affect × current depression × sleep duration	−**0.545**	−**1.036;** −**0.054**	**0.030**	−0.425	−0.970; 0.121	0.127
*Model 2c. Assessing differences between groups in morning affect, moderated by sleep quality* (*n* = 1,273)						
Intercept				**2.542**	**2.161; 2.922**	**<0.001**
Evening affect				**0.416**	**0.029; 0.803**	**0.035**
Depression Group (ref = never depressed)						
Past depression				−0.148	−1.080; 0.784	0.755
Current depression				−**1.723**	−**3.163;** −**0.282**	**0.019**
Sleep quality				**0.170**	**0.101; 0.240**	**<0.001**
Evening affect × sleep quality				−0.018	−0.091; 0.054	0.620
Depression group (ref = never depressed) × sleep quality						
Past depression × sleep quality				0.036	−0.142; 0.214	0.689
Current depression × sleep quality				**0.318**	**0.009; 0.627**	**0.044**
*Model 2d. Assessing differences between groups in morning affect, moderated by sleep duration* (*n* = 874)						
Intercept				**4.006**	**3.392; 4.620**	**<0.001**
Evening affect				0.547	−0.170; 1.264	0.134
Depression group (ref = never depressed)						
Past depression				−1.246	−2.880; 0.388	0.134
Current depression				1.079	−1.004; 3.162	0.308
Sleep duration				−0.067	−0.143; 0.009	0.082
Evening affect × sleep duration				−0.024	−0.112; 0.064	0.591
Depression group (ref = never depressed) × sleep duration						
Past depression × sleep duration				0.150	−0.051; 0.351	0.143
Current depression × sleep duration				−0.193	−0.450; 0.064	0.141
*Model 2e. Assessing differences between groups in morning affect, controlling for sleep* (*n* = 874)						
Intercept				**3.978**	**3.424; 4.531**	**<0.001**
Evening affect				**0.352**	**0.259; 0.444**	**<0.001**
Depression group (ref = never depressed)						
Past depression				−0.037	−0.288; 0.214	0.772
Current depression				−0.483	−0.889; −0.077	0.020
Sleep duration				−0.063	−0.131; 0.004	0.067

Notes. All significant effects related to hypotheses survived the Benjamini−Hochberg FDR-controlling (BH) procedure with a false discovery rate of 25%. Models 2a and 2b include person-mean centered PA or NA in the evening of the previous day, sleep quality/duration, the group status (past or current depression), and a three-way interaction between person-mean centered PA or NA in the evening, person mean sleep quality/duration, and the group status (past or current depression), where the never depressed group is the reference group. Models 2c and 2d include person-mean centered PA or NA in the evening of the previous day, person mean sleep quality/duration, the group status (past or current depression), and two two-way interactions between person-mean centered PA or NA in the evening and person mean sleep quality/duration, and between the group status and person mean sleep quality/duration. The interaction between person-mean centered PA or NA in the evening and the group status is not included in the model since it has been already reported in Table 2. Model 2e includes person-mean centered PA or NA in the evening of the previous day, person mean sleep quality/duration, the group status (past or current depression) without interaction terms. All models include the intercept.

*n = number of observations included in each model.

**Figure 2.**
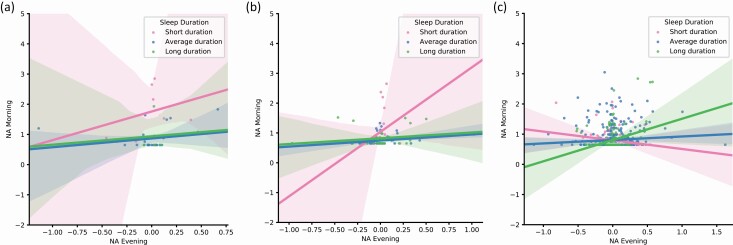
Associations between overnight affective inertia for negative affect (NA) and sleep duration for current (a), past (b), and never depressed groups (c).Note. NA evening affect is a mean-centered evening negative affect, and therefore it has both negative and positive values.

**Figure 3.**
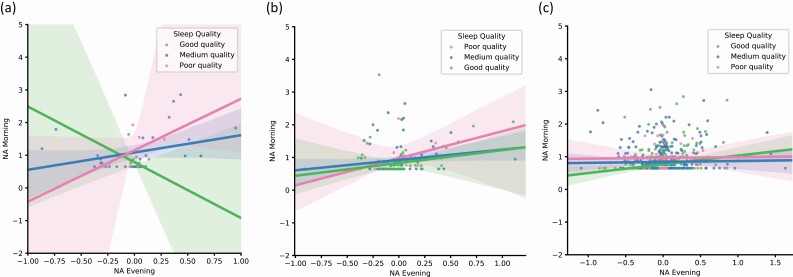
Associations between overnight affective inertia for negative affect (NA) and sleep quality for current (a), past (b), and never depressed groups (c).Note. Following sleep quality categories were chosen: 1–3 (poor), 4–5 (medium), 6–7 (good) sleep quality. Poor sleep quality category includes three points since there were extremely few observations with points 1 and 2. NA evening affect is a mean-centered evening negative affect, and therefore it has both negative and positive values.

To test significant three-way interactions for NA, we performed a set of subgroup analyses for sleep duration (Supplementary Table S2) and sleep quality (Supplementary Table S3). Additionally, we plotted the associations between morning NA and evening NA with three categories for sleep duration: short (<7 h), average (7–9 h), and long (>9 h) in Figure 2, a–c. We did the same for sleep quality with following categories: poor (1–3), medium (4–5), and good (6–7) sleep quality (Figure 3a-c). Results of the subgroup analyses revealed a positive association between overnight affective inertia and sleep duration in the never depressed group (*B* = 0.186) and a negative association in the current depression (*B* = –0.203) and past depression group (*B* = –1.600). This means that in the never depressed group, longer sleep duration was associated with higher overnight affective inertia, whereas in the current and past depression groups shorter sleep duration was associated with higher overnight affective inertia. In the current depression group, this negative association was not significant. For sleep quality, the differences between subgroups were in the same direction as for sleep quantity. However, all of the subgroup effects were non-significant.

We did not find significant three-way interactions between evening affect_*t−*1_, group status, and sleep quality or duration for PA. After removing the non-significant three-way interactions for PA (AIC changed from 3423 to 3411 for sleep quality and from 2416 to 2405 for sleep duration), the two-way interactions between current depression and sleep quality became significant (Model 2c). The two-way-interactions between current or past depression and sleep duration remained non-significant.

To further probe these significant two-way interaction effects for PA, we plotted the association between morning PA, evening PA, and sleep quality separately for the three groups in Supplementary Figure S2. The subgroup analysis revealed significant positive associations between sleep quality and morning PA in the past depression and never depressed groups separately (Supplementary Table S4). Although, no significant associations were found in the current depression group we observed steeper slopes in currently depressed individuals for sleep quality, compared to the never depressed group. Sleep duration was not differently associated with PA in different groups.

### Research question 3: The association between overnight and daytime affective inertia and the development of depression within a follow-up period

We did not find a significant interaction effect between group status (developing/not developing depression at the 12-months follow-up assessment) and evening affect_*t−*1_ (for neither NA nor PA), suggesting that overnight affective inertia did not predict the development of depression at the following 12 months (Table 4). After removal of the non-significant interaction (AIC changed from 2647 to 2642 for PA and from 431 to 427 for NA), being in the development group was significantly associated with higher morning NA but not PA, whereas a history of depression was not significantly associated with either morning NA or PA. For daytime affective inertia (*research question 4*), the interaction with group status was also non-significant (Table 4). The sensitivity analysis, in which we analyzed how overnight affective inertia predicts development of depression at the 12-months follow-up, did not display significant associations either. We found a significant association between daytime affective inertia and development of depression; however, this association was canceled after correcting for depressive symptoms at baseline (Table 5).

**Table 4.** Linear mixed-effects models estimated associations between morning and preceding evening affect (overnight affective inertia) and daytime affective inertia for PA and NA in depression development compared to the no depression groups

	Negative affect			Positive affect		
Fixed effects	*B*	95% CI	*p*-value	*B*	95% CI	*p*-value
***Overnight affective inertia***						
*Model 3a. Assessing differences between groups in overnight affective inertia* (*n* = 988)*						
Intercept						
Evening affect (autoregressive effect)	**0.274**	**0.138; 0.410**	**<0.001**	**0.352**	**0.264; 0.440**	**<0.001**
Development group	**0.124**	**0.049; 0.199**	**0.001**	−0.051	−0.298; 0.196	0.686
Depression history	0.025	−0.056; 0.105	0.546	0.108	−0.157; 0.372	0.423
Evening affect × development group	0.015	−0.254; 0.283	0.915	−0.017	−0.252; 0.219	0.887
*Model 3b. Assessing differences between groups in morning affect* (*n* = 988)						
Intercept						
Evening affect	**0.278**	**0.161; 0.395**	**<0.001**	**0.350**	**0.268; 0.431**	**<0.001**
Development group	**0.124**	**0.049; 0.200**	**0.001**	−0.050	−0.297; 0.196	0.687
Depression history	**0.025**	−**0.056; 0.105**	**0.546**	0.107	−0.157; 0.372	0.423
***Daytime affective inertia***						
*Model 3c. Assessing differences between groups in daytime affective inertia* (*n* = 13,344)						
Intercept	**0.805**	**0.779; 0.831**	**<0.001**	**3.595**	**3.512; 3.679**	**<0.001**
Affect_t_− _1_ (autoregressive effect)	**0.195**	**0.164; 0.225**	**<0.001**	**0.241**	**0.218; 0.265**	**<0.001**
Development group	**0.143**	**0.081; 0.205**	**<0.001**	−0.090	−0.281; 0.101	0.356
Depression history	0.032	−0.034; 0.097	0.339	−0.011	−0211; 0.189	0.915
Affect_*t*_− _1_ × development group	0.035	−0.036; 0.107	0.335	−0.001	−0.071; 0.052	0.767
*Model 3d. Assessing differences between groups in affect at previous time point* (*n* = 13,344)						
Intercept	**0.803**	**0.777; 0.829**	**<0.001**	**3.595**	**3.512; 3.679**	**<0.001**
Affect_*t*_− _1_	**0.240**	**0.223; 0.257**	**<0.001**	**0.240**	**0.218; 0.261**	**<0.001**
Development group	**0.145**	**0.084; 0.208**	**<0.001**	−0.090	−0.281; 0.101	0.355
Depression history	0.034	−0.032; 0.099	0.310	−0.011	−0.211; 0.190	0.915

Notes. All significant effects related to hypotheses survived the Benjamini−Hochberg FDR-controlling (BH) procedure with a false discovery rate of 25%. Model 3a includes person-mean centered PA or NA in the evening of the previous day and interaction between person-mean centered PA or NA in the evening of the previous day and the group status (group of individuals who developed depression during the follow-up period), where the non-depressed group is the reference group. Sleep variables are not included as they are assessed at the baseline and cannot be used in the follow-up analysis. For daytime affective inertia, Model 3c includes person-mean centered PA or NA at a previous time point (Affect_*t*_− _1_) instead of affect in the evening of the previous day. Both overnight and daytime affective inertia models were controlled for having history of depression at baseline. All models include the intercept.

*n = number of observations included in each model.

**Table 5.** Generalized linear models estimated associations between depression development and overnight or daytime affective inertia

	Depression development					
	Negative affect			Positive affect		
Fixed effects	*B*	95% CI	*p*-value	*B*	95% CI	*p*-value
***Overnight affective inertia*** (*n* = 383)*						
*Model 4a. Predicting development of depression by overnight affective inertia*						
Intercept	−**1.759**	−**2.056;** −**1.462**	**<0.001**	−**1.761**	−**2.059;** −**1.463**	**<0.001**
Overnight affective inertia	−0.255	−2.370; 1.861	0.814	−7.248	−33.419; 18.922	0.587
*Model 4b. Predicting development of depression by overnight affective inertia, controlling for baseline depressive symptoms*						
Intercept	−**3.957**	−**4.885;** −**3.029**	**<0.001**	−**3.957**	−**4.886;** −**3.028**	**<0.001**
Overnight affective inertia	0.067	−1.469; 1.603	0.932	−0.176	−15.561; 15.208	0.982
Depressive symptoms at baseline	**1.442**	**0.884; 2.000**	**<0.001**	**1.442**	**0.884; 2.001**	**<0.001**
***Daytime affective inertia*** (*n* = 404)						
*Model 4c. Assessing differences between groups in daytime affective inertia*						
Intercept	−**1.962**	−**2.262;** −**1.662**	**<0.001**	−**1.950**	−**2.248;** −**1.653**	**<0.001**
Daytime affective inertia	**2.361**	**0.012; 4.710**	**0.049**	2.313	−0.844; 5.469	0.151
*Model 4d. Predicting development of depression by daytime affective inertia, controlling for baseline depressive symptoms*						
Intercept	−1.302	−2.862; 0.258	0.102	−1.210	−2.783; 0.364	0.132
Daytime affective inertia	2.012	−1.006; 5.030	0.191	2.518	−1.436; 6.471	0.212
Depressive symptoms at baseline	−0.896	−2.081; 0.288	0.138	−0.964	−2.165; 0.238	0.116

Notes. All significant effects related to hypotheses survived the Benjamini−Hochberg FDR-controlling (BH) procedure with a false discovery rate of 25%. Models 4a and 4c includes overnight/daytime affective inertia estimates for PA or NA (random effects extracted from a multilevel model for overnight inertia). Models 4b and 4d were additionally controlled for depressive symptoms (SCL-90 score) at baseline. All models include the intercept.

*n = number of subjects included in each model.

## Discussion

In the current study, our aims were three-fold. First, we examined whether there is a difference in overnight affective inertia for positive (PA) and negative affect (NA) between groups of currently depressed, past depressed, and never depressed individuals. Second, we examined how sleep duration and sleep quality influence overnight affective inertia in these groups. Third, we tested whether differences in overnight affective inertia explained the development of depression over a period of 12-months. Finally, for comparison, we assessed aims one and three for daytime affective inertia as well.

Regarding the first aim, we found that individuals with current depression had significantly higher overnight affective inertia in NA compared to never depressed individuals. However, we did not find evidence that overnight affective inertia is dependent on depression status for PA. Since our sample consisted of only female participants and there is a substantial difference in emotion regulation between men and women [32], this could impact our results and the findings cannot be generalized to men. Due to lack of previous studies focusing on overnight affective inertia, comparison of our finding to the prior research is limited.

For daytime affective inertia, we did find that individuals in the past depression group had increased levels of negative, but not positive, affective inertia during the day, compared to never depressed individuals. These results are in line with the existing literature on daytime affective inertia in psychopathology, supporting the earlier reported finding that NA inertia is more strongly associated with depressive symptoms than PA inertia [3, 33, 34]. Surprisingly, we did not find the expected moderation effect for the current depression group. Given comparable estimates in both depression groups, it is possible that the limited number of assessments per individual, in combination with the relatively small sample size (*n* = 26), may have led to these non-significant results in the current depression group.

Regarding the second aim, higher overnight affective inertia for NA was associated with shorter sleep duration in both depression groups and lower sleep quality in the current depression group compared to the never depressed group. In line with our expectations, sleep quality and sleep duration differently influenced depressed and never depressed individuals with regards to the size of their overnight affective inertia for NA. However, we did not find such associations for PA. This is a novel finding and there are no studies, to our knowledge, that investigated this association, and thus can support or question this finding. This result should therefore be treated with caution until it is replicated.

After removal of the three-way interaction between evening PA, depression status and sleep characteristics, representing the second aim, the two-way interactions between sleep characteristics and depressive status to predict morning PA were examined. We found that poorer sleep quality was associated with lower morning PA in the currently depressed group, at least in the model correcting for sleep quality. Another study reported similar findings in a non-clinical female sample; they found that individuals experienced higher PA after a night of good quality sleep, and therefore, poor sleep may lead to decreased PA, which may partially explain how insomnia leads to depression [35]. Unlike our results, Bower and colleagues [18], as well as Bouwmans et al. [19], did not observe the moderation effect of a depression diagnosis on the association between sleep quality and PA. Similarly, another study did not observe the moderating effect of depressive symptoms on the association between sleep quality and PA [20]. This might be due to the fact that all above-mentioned studies used mean daily scores of affect for predicting next day’s affect instead of assessing daytime, evening and morning affect separately. Additionally, two of the studies used substantially smaller samples (96 [18] and 54 individuals [19]). Finally, unlike in our study, Bower and colleagues [19] used a cross-sectional measure of sleep quality over the past month, which is more prone to retrospective recall bias.

Regarding the final aim, we did not find evidence that overnight affective inertia is associated to depression diagnosis at 12 months follow-up. At the same time, results from the sensitivity analysis revealed significant association between day-time affective inertia for NA and development of depression at the 12-months follow up. However, this association became non-significant after controlling for baseline depressive symptoms. In contrast to our results, other studies found that greater day-time affective inertia of both PA and NA predicted the transition to clinical depression [36, 37]. In a study focusing on moment-to-moment transfer of PA (90-min lags), participants with higher momentary PA transfer in daily life had a lower level of depressive symptoms at follow-up [8]. Another study, however, reported that depressive symptoms predicted the strength of affective inertia 12 months later rather than the opposite, supporting the idea that affective inertia is a consequence rather than a precedent of depressive symptoms, at least in nonclinical populations [38]. Our findings also support this idea. However, due to the relatively long interval of one year, we cannot exclude that affective inertia is an early warning signal that increases relatively short before the onset of depression.

While we did not find evidence that affective inertia predicted depression development over the next year, after removing the interactions that assessed this aim, we did find that simply the morning negative affect at baseline predicted depression. Our study is not unique in this; one other study even found that assessing sad mood with a single-item scale can accurately predict relapse in recurrent depression [39]. Thus, despite the highlighted importance of patterns of emotional change that is argued by experts on affective dynamics [4, 33, 36, 37], more and more evidence accumulates suggesting that more complex emotion dynamic measures offer little added value over mean levels of PA and NA when predicting the development of depression [40–42].

As overnight affective inertia is an important but rather unexplored topic, it is interesting to highlight the observed differences between daytime and overnight affective inertia. We found that affective inertia for PA was much smaller during the day compared to affective inertia during the night, whereas it was smaller during the night for NA than during the day but still different from zero. This provides some clues to answer the question on “how to treat the night,” which is an often-encountered problem in ESM research. Various studies have treated subsequent days of ESM as totally uncorrelated, considering night as a reset point for affect [43] either by including as many missing lags as fit in a night (e.g. six missing values for six lags of 90 min) or splitting the dataset and analyzing the days separately [44, 45]. Another approach was to treat the night as the equivalent to one daytime lag [45, 46]. Based on the results of the current study, we do not recommend treating the night as a reset point, nor as a single lag, and rather differently for PA and NA. However, the exact number of lags should be further explored separately for PA and NA in future studies across different samples.

The present study had several important strengths, such as its prospective nature, availability of a depression diagnosis, large total sample size, and multilevel data analysis. However, it has several limitations as well. First, all available participants were female adults which limits generalizability of our findings to women only. A second limitation is the size of the current depression group (*n* = 26) for hypothesis 1 and 2. The study may have been underpowered to detect differences between this group and the other groups. Third, a worth-mentioning limitation is in the duration of the ESM observations. Only five days of ESM observations were available. Therefore, the obtained within-individual changes might be not as reliable as from longer ESM observations (i.e. weeks). This also hindered calculation of overnight affective inertia and decreased its variation per person since only a few observations per person were available from the five days of the ESM data. Finally, this dataset was primarily collected for twin research and not specifically for sleep research, therefore we had limited information on insomnia among the participants and no information on sleep medication use. Additionally, due to the same reason, we only had self-reported sleep data in this dataset. Objective sleep data would have been an asset, especially for sleep duration, in addition to self-reported sleep data, as objective and subjective sleep quality might be two different concepts and each possesses a unique value [47].

One important point that was not covered in the current study is possibly low variation of NA within individuals across nights. This should be taken into account in future studies, possibly by designing different NA measures, as limited variation in affect characteristics could reduce the ability to identify associations. Another interesting point that should be further investigated is emotion specificity within PA and NA sum scores. Previously, researchers found that the association between daytime inertia and psychopathology might show domain specificity. Specifically, symptoms of depression were more strongly associated with affective inertia in feeling suspicious, down, and listless as they are considered to cover the depression domain [48]. However, such specificity of affective states was not investigated for overnight affective inertia. Exploring this question might extend our understanding of affect dynamics in psychopathology. Finally, the possibility of cumulative effects of poor sleep and high overnight inertia over multiple consecutive nights, and their potential to increase the risk for depression, is an interesting topic for future studies with longer time series.

In conclusion, our study investigated the associations between overnight and daytime affective inertia and depression, additionally taking into account sleep quality and duration. Our findings suggest that overnight NA inertia is larger in depressed compared to non-depressed individuals, and that poor sleep even exacerbates these differences, whereas good sleep negates these differences. In addition, emotional dynamics of NA appear to be more prominently disturbed in depression compared to PA. This study makes an important contribution to the literature by enhancing the understanding of complex emotion dynamics more specifically, overnight affective inertia, as well as simpler measures such as mean levels of PA and NA in relation to depression and its development over 12 months.

## Supplementary Material

zsab129_suppl_Supplementary_Materials
