# Supplementary material for: Overnight affective dynamics and sleep characteristics as predictors of depression and its development in women
Source: Sleep. 2021 May 20;44(10):zsab129. doi: 10.1093/sleep/zsab129 (PMC8503829; doi:10.1093/sleep/zsab129)
Supplement: zsab129_suppl_Supplementary_Materials [file zsab129_suppl_supplementary_materials.docx]

**Supplementary Materials**

**Overnight affective dynamics and sleep characteristics as predictors of depression and its development in women**

Olga Minaeva^1^, Sandip V. George^1^, Anna Kuranova^1^, Nele Jacobs^2,3^, Evert Thiery^4^, Catherine Derom^5,6^, Marieke Wichers^1^, Harriëtte Riese^1^, Sanne H. Booij^1,7^

^1^University of Groningen, University Medical Center Groningen, Department of Psychiatry, Interdisciplinary Center for Psychopathology and Emotion regulation, Groningen, the Netherlands

^2^Maastricht University, Department of Psychiatry and Neuropsychology, School of Mental Health and Neuroscience (MHeNS), Maastricht, the Netherlands

^3^Open University of the Netherlands, Faculty of Psychology, Heerlen, the Netherlands

^4^Ghent University, Ghent University Hospital, Department of Neurology Ghent, Belgium

^5^KU Leuven, University Hospital Leuven, Centre of Human Genetics, Leuven, Belgium

^6^Ghent University, Ghent University Hospital, Department of Obstetrics and Gynecology, Ghent, Belgium

^7^Lentis, Center for Integrative Psychiatry, Groningen, the Netherlands

*Corresponding author:

Olga Minaeva

University of Groningen, University Medical Center Groningen, Department of Psychiatry (CC72), P.O. Box 30.001, 9700 RB Groningen, the Netherlands. [o.minaeva@umcg.nl](mailto:o.minaeva@umcg.nl)

**Contents:**

- Supplementary material 1. Detailed description of the models
- Supplementary material 2.
  - Table S1. Associations between evening NA and morning NA in three different groups
  - Table S2. Associations between morning NA and evening NA, moderated by sleep duration in three different groups
  - Table S3. Associations between morning NA and evening NA, moderated by sleep quality in three different groups
  - Table S4. Associations between morning PA and evening PA, controlled for sleep quality in three different groups
- Supplementary material 3
  - Figure S1. Daytime affective inertia for negative affect (NA) for current, past, and never depressed groups.
  - Figure S2. Associations between morning PA and sleep quality for current (a), past (b), and never depressed groups (c).

**Supplementary material 1. Detailed description of the models**

In our first hypothesis we investigate how the overnight affective inertia varies across individuals with differing histories of depression. Individuals are categorized into three groups based on their depression status, namely individuals with a history of depression but are not currently depressed, currently depressed individuals, and individuals without a history of depression at baseline. We consider two separate sub-models to study this hypothesis. The first checks how the affect variable at night and the interaction between night affect and depression status predict the affect in the morning. It is described by the following equation:

$$M{affect}_{dij}= \beta_{0ij}+\beta_{1ij}\left( {Eaffect}_{d-1ij}-{<Eaffect>}_{i} \right)+ \beta_{2ij}D_{i}+ \beta_{3ij}\left( D_{i}*\left( {Eaffect}_{d-1ij}-<Eaffect>_{i} \right) \right)+ \varepsilon_{ij}$$

*Eq 1*

Here d represents the day, i is the individual level and j is the twin level. Maffect_dij_ and Eaffect_dij_ are the morning and evening affect respectively on day d for individual I and twin pair j. D_i_ is a categorical variable that denotes the depression status for individual i. Quantities within <> represent the person level mean of that duration.

Among the coefficients, $\beta_{1ij}$ quantifies the overnight affective inertia, $\beta_{2ij}$ quantifies how well depression status predicts morning affect, and $\beta_{3ij}$ quantifies the interaction effect of person-mean centered night affect and depression status on morning affect. Hence, the interaction term $\beta_{3ij}$ provides the association between overnight affective inertia and depression status.

Our second sub-model, controls for sleep variables, namely the sleep quality rated on a Likert scale and the sleep duration measured in hours. Both sleep variables are person-mean centered, and the mean values are added as a predictor in the model. The person-centered sleep variable quantifies the within person association between sleep and morning affect, whereas the person mean of the sleep variable quantifies the person level effect that the average has on the morning affect. The model can be written as

$$M{affect}_{dij}= \beta_{0ij}+\beta_{1ij}\left( {Eaffect}_{d-1ij}-{<Eaffect>}_{i} \right)+ \beta_{2ij}D_{i}+ \beta_{3ij}\left( D_{i}*\left( {Eaffect}_{d-1ij}-{<Eaffect>}_{i} \right) \right)+\beta_{4ij}\left( SQ_{dij}-<SQ>_{i} \right)+\beta_{5ij}<SQ>_{i}+\beta_{6ij}\left( SD_{dij}-<SD>_{i} \right)+\beta_{7ij}<SD>_{i}+ \varepsilon_{ij}$$

*Eq 2*

In addition to Eq1, the terms, SQ and SD refer to sleep quality and duration respectively.

In this case $\beta_{4ij}$ quantifies how well the sleep quality at night predicts the affect the next morning, $\beta_{5ij}$refers to how well the mean sleep quality of the individual predicts the affect the next morning, $\beta_{6ij}$ quantifies how well the sleep duration at night predicts the affect the next morning and $\beta_{7ij}$refers to how well the mean sleep duration of the individual predicts the affect the next morning.

An interesting comparison for the magnitude of overnight affective inertia is provided by the daytime autocorrelation. This is investigated using the multilevel model given using

$${affect}_{tij}= \beta_{0ij}+\beta_{1ij}\left( {affect}_{t-1ij}-{<affect>}_{i} \right)+ \beta_{2ij}D_{i}+ \beta_{3ij}\left( D_{i}*\left( {affect}_{t-1ij}-<affect>_{i} \right) \right)+ \varepsilon_{ij}$$

*Eq 3*

Here, t refers to the time at which the quantifier (affect) is measured. $\beta_{1ij}$ now quantifies the autocorrelation between the affect at time t and at time t-1. $\beta_{2ij}$ quantifies how well the affect is predicted by the depression status and $\beta_{3ij}$ quantifies how depression status affects the autocorrelation.

Our second hypothesis we investigate the influence of sleep duration and sleep quality on overnight affective inertia and if there exists a variation in this influence between groups based on depression status. This is investigated using a multilevel model having the morning affect as the outcome variable and containing sleep variables, depression status and night affect, and interactions between them as predictor variables. We specifically seek how the person level mean values of the sleep variables influence overnight affective inertia in groups. We make separate models for sleep quality and sleep duration, and they are presented below.

$$M{affect}_{dij}= \beta_{0ij}+\beta_{1ij}\left( {Eaffect}_{d-1ij}-{<Eaffect>}_{i} \right)+ \beta_{2ij}D_{i}+ \beta_{3ij}\left( D_{i}*\left( {Eaffect}_{d-1ij}-{<Eaffect>}_{i} \right) \right)+\beta_{4ij}<SQ>_{i} +\beta_{5ij}\left( D_{i}*<SQ>_{i} \right)+ \beta_{6ij}\left( (Eaffect_{d-1ij}-{<Eaffect>}_{i})*<SQ>_{i} \right)+\beta_{7ij}((Eaffect_{d-1ij}-{<Eaffect>}_{i})*<SQ>_{i}*D_{i}) + \varepsilon_{ij}$$

Eq 4

$M{affect}_{dij}= \beta_{0ij}+\beta_{1ij}\left( {Eaffect}_{d-1ij}-{<Eaffect>}_{i} \right)+ \beta_{2ij}D_{i}+ \beta_{3ij}\left( D_{i}*\left( {Eaffect}_{d-1ij}-{<Eaffect>}_{i} \right) \right)+\beta_{4ij}<SD>_{i} +\beta_{5ij}\left( D_{i}*-<SD>_{i} \right)+ \beta_{6ij}\left( (Eaffect_{d-1ij}-{<Eaffect>}_{i})*<SD>_{i} \right)+\beta_{7ij}((Eaffect_{d-1ij}-{<Eaffect>}_{i})*<SD>_{i}*D_{i}) + \varepsilon_{ij}$

Eq 5

Here, $\beta_{5ij}$quantifies the extent to which the interaction between mean sleep quality/duration and depression status predicts the morning affect, $\beta_{6ij}$ quantifies the extent to which the interaction between mean sleep quality/duration and night affect predict the morning affect and finally $\beta_{7ij}$ gives the extend to which the three way interaction effects between night affect, sleep quality/duration and depression status predicts the morning affect.

We then consider two models with separate two-way interactions, namely the interaction of night affect and depression status with the sleep variables. This leaves us with Eq 3 and 4 after removing the terms corresponding to $\beta_{3ij}$ and $\beta_{7ij}$. These are listed below

$$M{affect}_{dij}= \beta_{0ij}+\beta_{1ij}\left( {Eaffect}_{d-1ij}-{<Eaffect>}_{i} \right)+ \beta_{2ij}D_{i}+\beta_{4ij}<SQ>_{i} +\beta_{5ij}\left( D_{i}*<SQ>_{i} \right)+ \beta_{6ij}\left( Eaffect_{d-1ij}*<SQ>_{i} \right)$$

Eq 6

$$M{affect}_{dij}= \beta_{0ij}+\beta_{1ij}\left( {Eaffect}_{d-1ij}-{<Eaffect>}_{i} \right)+ \beta_{2ij}D_{i}+\beta_{4ij}<SD>_{i} +\beta_{5ij}\left( D_{i}*<SD>_{i} \right)+ \beta_{6ij}\left( Eaffect_{d-1ij}*<SD>_{i} \right)$$

Eq 7

Finally, we use a combined model using both sleep quality and sleep duration together, with only two-way interactions, given as

$$M{affect}_{dij}= \beta_{0ij}+\beta_{1ij}\left( {Eaffect}_{d-1ij}-{<Eaffect>}_{i} \right)+ \beta_{2ij}D_{i}+\beta_{3ij}<SQ>_{i} +\beta_{4ij}\left( D_{i}*<SQ>_{i} \right)+ \beta_{5ij}\left( Eaffect_{d-1ij}*<SQ>_{i} \right)+\beta_{6ij}<SD>_{i} +\beta_{7ij}\left( D_{i}*<SD>_{i} \right)+ \beta_{8ij}\left( Eaffect_{d-1ij}*<SD>_{i} \right)$$

Eq 8

For our third hypothesis, we investigate how the autocorrelation predicts the development of depression at follow up. In order to investigate this question, we construct a multilevel model, inverting the predictor and outcome variables. Hence, we examine how the development of depression predicts the morning affect, and how it interacts with the night affect. For this we construct a categorical variable, Dev, which takes different values depending on whether the individual develops depression between baseline and follow up, or not. We exclude all individuals diagnosed with depression at baseline for this analysis. The multilevel model to explore this is constructed as

$$M{affect}_{dij}= \beta_{0ij}+\beta_{1ij}\left( {Eaffect}_{d-1ij}-{<Eaffect>}_{i} \right)+ \beta_{2ij}{Dev}_{i}+ \beta_{3ij}\left( {Dev}_{i}*\left( {Eaffect}_{d-1ij}-<Eaffect>_{i} \right) \right)+ \varepsilon_{ij}$$

Eq 9

Here, $\beta_{2ij}$ quantifies how much the development groups predict the morning affect, and $\beta_{3ij}$ quantifies how much the morning affect is predicted by the extend of interaction between person-mean centered night affect and depression development.

As with the first hypothesis we check how the affect measured at a particular time during the day is predicted by the mean-centered affect measured at the previous time point, the depression development status and the interaction between these two.

$${affect}_{tij}= \beta_{0ij}+\beta_{1ij}\left( {affect}_{t-1ij}-{<affect>}_{i} \right)+ \beta_{2ij}{Dev}_{i}+ \beta_{3ij}\left( {Dev}_{i}*\left( {affect}_{t-1ij}-<affect>_{i} \right) \right)+ \varepsilon_{ij}$$

Eq 10

Here, t refers to the time at which the quantifier (affect) is measured. $\beta_{1ij}$ now quantifies the autocorrelation between the affect at time t and at time t-1. $\beta_{2ij}$ quantifies how well the affect is predicted by the development groups and $\beta_{3ij}$ quantifies how the extend to which the autocorrelation varies within the development groups.

**Supplementary material 2.**

**Table S1. Associations between evening NA and morning NA in three different groups**

|  | B | 95% CI | p-value |
| --- | --- | --- | --- |
| *Non-depressed group* (n=1070) | | | |
| Intercept | **0.797** | **0.771; 0.822** | **<0.001** |
| Evening affect | **0.167** | **0.056; 0.279** | **0.003** |
| *After controlling for sleep duration and quality* (n=723) | | | |
| Intercept | **0.909** | **0.690; 1.128** | **<0.001** |
| Evening affect | 0.132 | -0.032; 0.297 | 0.114 |
| Sleep quality | **-0.041** | **-0.065; -0.018** | **<0.001** |
| Sleep duration | 0.014 | -0.009; 0.038 | 0.232 |
| *Past depression group* (n=181) | | | |
| Intercept | **0.879** | **0.802; 0.956** | **<0.001** |
| Evening affect | **0.379** | **0.096; 0.661** | **0.009** |
| *After controlling for sleep duration and quality* (n=114) | | | |
| Intercept | **1.062** | **0.539; 1.586** | **<0.001** |
| Evening affect | **1.791** | **0.201; 3.380** | **0.028** |
| Sleep quality | -0.042 | -0.112; 0.028 | 0.205 |
| Sleep duration | 0.001 | -0.064; 0.065 | 0.993 |
| *Current depression group* (n=61) | | | |
| Intercept | **0.993** | **0.807; 1.178** | **<0.001** |
| Evening affect | **0.614** | **0.100; 1.129** | **0.021** |
| *After controlling for sleep duration and quality* (n=39) | | | |
| Intercept | **3.141** | **1.397; 4.884** | **0.001** |
| Evening affect | **0.502** | **0.086; 0.919** | **0.021** |
| Sleep quality | -0.193 | -0.391; 0.004 | 0.054 |
| Sleep duration | -0.151 | -0.309; 0.008 | 0.061 |

**Table S2. Associations between morning NA and evening NA, moderated by sleep duration in three different groups**

|  | B | 95% CI | p-value |
| --- | --- | --- | --- |
| *Non-depressed group* (n=729) | | | |
| Intercept | **0.715** | **0.524; 0.907** | **<0.001** |
| Evening affect | **-1.337** | **-2.564; -0.111** | **0.033** |
| Sleep duration | 0.011 | -0.013; 0.035 | 0.365 |
| Evening Affect*Sleep duration | **0.186** | **0.035; 0.338** | **0.016** |
| *Past depression group* (n=114) | | | |
| Intercept | **1.257** | **0.737; 1.776** | **<0.001** |
| Evening affect | **14.641** | **3.404; 25.879** | **0.012** |
| Sleep duration | -0.052 | -0.123; 0.019 | 0.133 |
| Evening Affect*Sleep duration | **-1.600** | **-2.970; -0.231** | **0.023** |
| *Current depression group* (n=40) | | | |
| Intercept | **1.949** | **0.647; 3.252** | **0.006** |
| Evening affect | 2.175 | -2.477; 6.827 | 0.339 |
| Sleep duration | -0.111 | -0.271; 0.049 | 0.163 |
| Evening Affect*Sleep duration | -0.203 | -0.792; 0.386 | 0.478 |

**Table S3. Associations between morning NA and evening NA, moderated by sleep quality in three different groups**

|  | B | 95% CI | p-value |
| --- | --- | --- | --- |
| *Non-depressed group* (n=1042) | | | |
| Intercept | **1.065** | **0.946; 1.183** | **<0.001** |
| Evening affect | -0189 | -0.704; 0.326 | 0.471 |
| Sleep quality | **-0.049** | **-0.071; -0.028** | **<0.001** |
| Evening Affect*Sleep quality | 0.069 | -0.030; 0.168 | 0.171 |
| *Past depression group* (n=172) | | | |
| Intercept | **1.138** | **0.793; 1.482** | **<0.001** |
| Evening affect | 1.014 | -0.584; 2.611 | 0.211 |
| Sleep quality | -0.050 | -0.123; 0.024 | 0.163 |
| Evening Affect*Sleep quality | -0.127 | -0.449; 0.194 | 0.433 |
| *Current depression group* (n=59) | | | |
| Intercept | **1.876** | **1.265; 2.587** | **<0.001** |
| Evening affect | 3.184 | -0.211; 6.579 | 0.065 |
| Sleep quality | -0.197 | -1.161; 0.767 | 0.234 |
| Evening Affect*Sleep quality | -0.603 | -1.414; 0.208 | 0.140 |

**Table S4. Associations between morning PA and evening PA, controlled for sleep quality in three different groups**

|  | B | 95% CI | p-value |
| --- | --- | --- | --- |
| *Non-depressed group* (n=1042) | | | |
| Intercept | **2.552** | **2.174; 2.929** | **<0.001** |
| Evening affect | **0.300** | **0.212; 0.388** | **<0.001** |
| Sleep quality | **0.169** | **0.100; 0.237** | **<0.001** |
| *Past depression group* (n=172) | | | |
| Intercept | **2.258** | **1.434; 3.082** | **<0.001** |
| Evening affect | **0.410** | **0.233; 0.587** | **<0.001** |
| Sleep quality | **0.238** | **0.063; 0.413** | **0.012** |
| *Current depression group* (n=59) | | | |
| Intercept | 0.977 | -0.519; 2.472 | 0.193 |
| Evening affect | 0.356 | -0.035; 0.747 | 0.073 |
| Sleep quality | 0.459 | -1.584; 2.502 | 0.214 |

**Supplementary material 3**

**
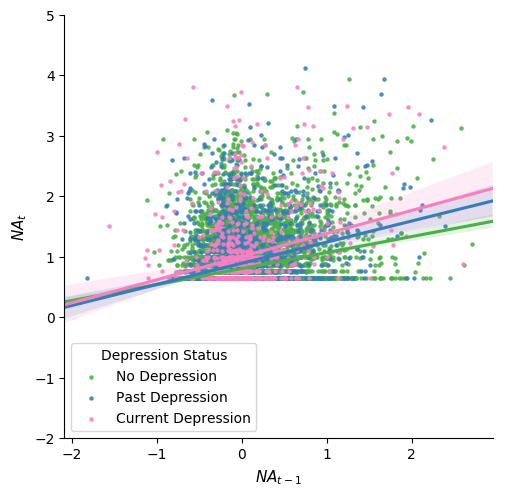
**

**Figure S1. Daytime affective inertia for negative affect (NA) for current, past, and never depressed groups.**


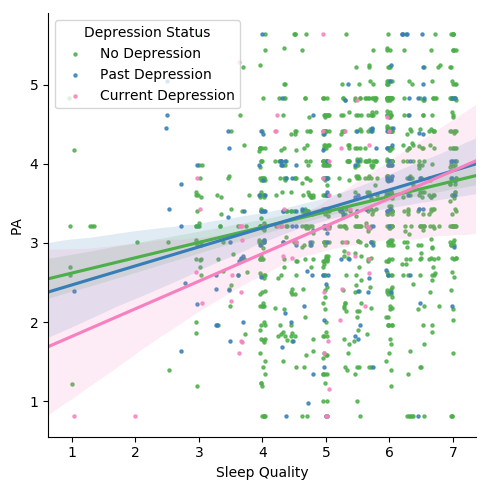


**Figure S2. Associations between morning PA and sleep quality for current (a), past (b), and never depressed groups (c).**
